# Population Specific and Up to Date Cardiovascular Risk Charts Can Be Efficiently Obtained with Record Linkage of Routine and Observational Data

**DOI:** 10.1371/journal.pone.0056149

**Published:** 2013-02-14

**Authors:** David Faeh, Julia Braun, Kaspar Rufibach, Milo A. Puhan, Pedro Marques-Vidal, Matthias Bopp, Swiss National Cohort (SNC)

**Affiliations:** 1 Institute of Social and Preventive Medicine (ISPM), University of Zurich, Zurich, Switzerland; 2 Department of Epidemiology, Johns Hopkins Bloomberg School of Public Health, Baltimore, Maryland, United States of America; 3 Institute of Social and Preventive Medicine (IUMSP), Lausanne University Hospital, Lausanne, Switzerland; University of Sao Paulo, Brazil

## Abstract

**Background:**

Only few countries have cohorts enabling specific and up-to-date cardiovascular disease (CVD) risk estimation. Individual risk assessment based on study samples that differ too much from the target population could jeopardize the benefit of risk charts in general practice. Our aim was to provide up-to-date and valid CVD risk estimation for a Swiss population using a novel record linkage approach.

**Methods:**

Anonymous record linkage was used to follow-up (for mortality, until 2008) 9,853 men and women aged 25–74 years who participated in the Swiss MONICA (MONItoring of trends and determinants in CVD) study of 1983–92. The linkage success was 97.8%, loss to follow-up 1990–2000 was 4.7%. Based on the ESC SCORE methodology (Weibull regression), we used age, sex, blood pressure, smoking, and cholesterol to generate three models. We compared the 1) original SCORE model with a 2) recalibrated and a 3) new model using the Brier score (BS) and cross-validation.

**Results:**

Based on the cross-validated BS, the new model (BS = 14107×10^−6^) was somewhat more appropriate for risk estimation than the original (BS = 14190×10^−6^) and the recalibrated (BS = 14172×10^−6^) model. Particularly at younger age, derived absolute risks were consistently lower than those from the original and the recalibrated model which was mainly due to a smaller impact of total cholesterol.

**Conclusion:**

Using record linkage of observational and routine data is an efficient procedure to obtain valid and up-to-date CVD risk estimates for a specific population.

## Introduction

The SCORE (Systematic COronary Risk Evaluation) project from the European Society of Cardiology (ESC) pooled a dozen of prospective cohorts from European countries. [1] The aim was to provide a method to estimate absolute risk for fatal cardiovascular disease (CVD) based on major CVD risk factors. [1] The result is a clinically useful and evidence-based tool for risk prediction widely used in clinical practice. [2] However, for several reasons, CVD risk estimation may be misleading. First, there is substantial variation in the prevalence of CVD risk factors, mortality and trends of CVD across populations and with respect to the relationship between CVD risk factors and risk of death.[1], [3]–[5] Such differences are not fully taken into account by pooled data originating from different countries, even if separate models for high and low risk countries are provided for SCORE. [1], [6] Second, there is long latency between data collection and the generation of risk scores and their use by physicians. As a consequence, risk formulas derived from persons having died, for example, 40 years ago, are used in today’s context and changes in risk factors in the population and medical progress are not taken into account. Third, there has been a dramatic decrease in CVD mortality in most countries with a concomitant increase in life expectancy. [3], [7] These demographic changes should be reflected in the risk scores, e.g. by shifting the prediction age classes towards older ages. Currently, the SCORE risk function is restricted to a maximum age of 65 years. [1].

Unfortunately, most European countries - particularly those at low risk - do not have cohort studies necessary to provide country specific and up-to-date risk functions. Therefore, these countries use risk factor coefficients defining the “individual hazardousness” from “foreign” populations. Previous attempts to adapt the SCORE were limited by the fact that mortality did not stem from the population that provided CVD risk factors (health survey) but from the general population (death registry).[8]–[10] This precludes considering changes in the association between risk factors and their combination and the risk of death.

In this study we aimed at adapting the SCORE method in a way that it provides up-to-date and population specific valid estimates by using available routine and observational data. For this purpose, we combined data from the Swiss National Cohort (SNC, a record linkage of data from the census, death and migration registries) with data from a CVD health survey. We aimed at comparing the original SCORE model (low-risk countries) with a recalibrated version of the original SCORE model and with a new model including coefficients for risk factors derived from our linked database. In Switzerland, CVD mortality has substantially decreased over the past three decades, resulting in very low rates compared to other countries. [3], [7] In contrast, the prevalence of major CVD risk factors remained relatively stable or only slightly declined, but is still lower than in most other countries. [3], [4].

## Methods

### Population

We used data from the Swiss MONICA population survey. MONICA is an international project of the World Health Organization (WHO) aimed at monitoring trends and determinants in CVD. [11] In Switzerland, the study has been conducted in the cantons Vaud/Fribourg and Ticino and in three waves during period 1983–92 in men and women aged 25–74. Of the initially sampled persons, between 54% and 78% participated in the study.[12]–[14] Lack of mortality follow-up was overcome by an anonymous record linkage with the SNC. [14], [15] Approval (Nr. 13/06) was obtained from the Ethics Committee of the Canton of Zurich. Details of the study sample are given in Table 1.

**Table 1:** Characteristics (counts, means and proportions) of the Swiss MONICA study sample and the ESC SCORE sample.

	MONICA		ESC SCORE*	
	Men	Women	Men	Women
Countries	Switzerland		3 European*	
Recruitment years	1983–1992		1974–1988	
Participation rate (%)	54–78		36–75	
95th centile of follow-up (years)	23.9		10.1–13.7	
Participants with mortality follow-up (n)	4784	4662	37183	31598
Mean age at baseline; standard deviation (years)	47.0; 11.3	47.2; 11.5	*NR*	
Age range at baseline (years)	25–74		19–80	
Deaths				
Cardiovascular disease (% of all deaths)	29.9	28.0	*NR*	
Coronary heart disease (n)	143	53	*NR*	
Coronary heart disease (% of all CVD)	52.8	35.1	47–77	38–70
Non-coronary CVD (n)	128	98	*NR*	
All causes	906	539	*NR*	
Deaths in persons aged ≥65 years (% of all deaths)	67.0	74.0	*NR*	
Current smoker (%)	32.2	24.8	46–54	12–22
Systolic blood pressure (mmHg)	132.0	126.0	132–136	120–133
Mean total Cholesterol (mmol/l)	6.2	6.0	5.6–6.0	5.5–6.1
Mean total-to-HDL-cholesterol ratio	5.7	4.4	4.4–4.8	3.8–4.0

* “Low-risk” countries: Belgium (n = 10,641), Italy (n = 53,439), Spain (n = 4,701).

HDL: High-density lipoprotein.

NR: not reported.

MONICA: MONItoring of trends and determinants in CArdiovascular disease.

### Record Linkage Procedure

In order to determine vital status of MONICA participants, we used an anonymous record linkage with the SNC. The SNC encompasses all residents of Switzerland included in the national censuses of 1990 and 2000 (6.8 and 7.3 million, respectively). As described elsewhere, deterministic and probabilistic methods were used to link anonymised census, death and emigration records. [15] In a second phase, SNC information was linked to MONICA data. This additional record linkage was also entirely anonymous. The linkage was based on procedures including all potential identification variables, i.e. variables available in MONICA and in the SNC. The minimal required information for a promising record linkage was sex, exact date of birth and place of residence (community). Additional identification variables were nationality, marital status, educational category and profession. [14].

As the same individual could be sampled in more than one MONICA wave, all participants with identical sex/date of birth/place of residence were checked for repeated sampling, with a plausibility test based on profession, body height, body weight and blood pressure.

Record linkage between MONICA and SNC was performed stepwise, with satisfactorily linked individuals excluded from succeeding steps (MONICA III, which was conducted in 1992/3, is not involved in the steps 2 and 5, because no deaths occurred before 1990 among participants):

1. MONICA community  = 1990 census community of residence
2. Participants of MONICA I, II: MONICA community = community in mortality records (only deaths occurred before 1990 census)
3. MONICA community  = 1985 community of residence (based on 1990 census)
4. MONICA community  = 2000 or 1995 community of residence (based on 2000 census)
5. Participants of MONICA I, II: linkage with other community based on mortality statistics (only deaths occurred before the 1990 census)
6. linkage with other community of 1990 census in the same canton
7. linkage with community of 1990 census in another canton
8. manual control and optimization (check of remaining unlinked MONICA participants for potential partner records in the 1990 census and the 1984–90 mortality records. Typically these records showed discordances regarding date of birth, place of residence and occupation, which prevented automated record linkage. Still, considering all available information and potential alternative links, it was inferred that the records referred to the same individual)

MONICA wave, region of residence (Vaud/Fribourg, Ticino), age, sex, nationality (Swiss or foreign), marital status and educational level (mandatory, upper secondary, tertiary, university education) were included as independent variables in a logistic regression model to analyse the odds for linkage failures or loss to follow-up between the censuses of 1990 and 2000.

Of the eligible 10,160 MONICA participants, 97.8% could be linked to a census (9,737 in 1990 and 8,749 in 2000), mortality (1,526 for period 1984–2008) and/or emigration record (320 for period 1990–2008). Eighty-three participants of the 1992 wave of MONICA could only be linked to the preceding 1990 census but not to a subsequent census, mortality or emigration record, thus leaving 9,853 individuals for survival analysis. Loss to follow-up between 1990 and 2000 amounted to 4.7%. Since there was no census at the end of the study, loss to follow-up after the 2000 census could not be determined. Hence, all 7,854 individuals linked to the 2000 census but not to a succeeding death or emigration record were considered as being alive. [14].

### Exposure and Outcome

Measurements and blood sampling procedures were described earlier. [12], [13] We defined smoking as current smoking irrespective of the number of cigarettes. Non-smokers included former and never smokers. Systolic blood pressure was computed as the mean of two or three successive measurements. Outcome data (death with underlying cause) was derived from the Swiss national death registry; this information is included in the SNC. [15] Fatal CVD events were defined according to the International Classification of Diseases (ICD) revisions 8 (ICD-8∶390–458, until 1994) and 10 (ICD-10: I00-I99, since 1995). For recalibration, we divided CVD into coronary heart disease (CHD; ICD-8∶410–414, ICD-10: I20–I25) and non-coronary CVD, according to the literature. [1].

### Survival Analysis

Risk score calculation based on Weibull proportional hazards regression models. The corresponding hazard function is:
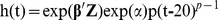
where **Z** contains the values of the covariates and **β** is the vector of the corresponding coefficients. The other parameters, *α* and p, are the logarithm of the intercept and the scale parameter, respectively. As in, [1] the time variable t in the hazard function is the person's age at either 1) date of death (from mortality records) or 2) the censoring time point, i.e., 12/31/2008.

Individual 10 year risk scores were obtained using three approaches: 1) based on the original SCORE coefficients as described in [1]; 2) based on a recalibration of the original SCORE model with the methodology described in [16]; 3) based on new coefficients derived from Weibull regression models applied to our data.

### Models

We used the original SCORE with its coefficients for low-risk countries (Belgium n = 10,641; Italy, n = 53,439; Spain, n = 4,701) as reference. [1] We recalibrated the original model [1] by fitting two new Weibull regression models (one for CHD and one for non-coronary CVD) to our data. In these models, only the parameters α and p were estimated, the remaining coefficients from the two original models being fixed and included as an offset. This corresponds to recalibration as described in, [16] where the values of the covariates and their coefficients serve as offset and the intercept and remaining parameters are shifted to better represent the characteristics of the actual population. Additionally, two completely new models were calculated with current smoking, systolic BP (mmHg) and total cholesterol or, alternatively, cholesterol ratio (total-to-HDL-cholesterol) as covariates. To keep these two models as simple as possible, we calculated only one joint model for CVD deaths and omitted the partition into CHD and non-coronary CVD deaths. We took this somewhat arbitrary decision to circumvent misclassification of deaths, which could potentially result in information bias. As shown in the original SCORE sample, there were large variations in the proportion of non-coronary CVD cases, possibly due to cultural differences in assignment of causes of death. [1] In Switzerland, the extent to which CHD and non-coronary CVD deaths decreased over the past decades was similar, suggesting similar pathophysiological mechanisms and comparable progress in medical treatment. [7].

### Model Comparison

In order to compare the predictive abilities of the different risk scores (original, recalibrated, new), we calculated the Brier score. [17] The Brier score measures the mean squared difference between the risk score and the actual outcome. The lower this deviation, the better the respective risk prediction model. The Brier score accounts for both discrimination (i.e. correct classification in different outcome groups) and calibration (agreement of the predictions with the true risk). [16] This is a clear advantage over other common methods for prediction assessment, especially the receiver operating characteristic (ROC) curve and the net reclassification improvement (NRI) which both focus on discrimination. Discrimination is important in a diagnostic setting where the classification of individuals into different groups is the main interest. However, in a prognostic setting, where individuals probabilities of future events are the main goal, calibration is of paramount importance and should not be ignored. [18] Moreover, the Brier score is easy to interpret and is not dependent on an arbitrary definition of thresholds for the classification of individual risk scores to different risk groups.

As the Brier score would be too optimistic in the case where model fitting and assessment are performed using the same data (i.e. for the recalibrated and the new model), we used leave-one-out cross-validation: after one person is excluded from the data set, the Weibull regression model is re-fitted to the remaining data, and the risk score is then estimated for the person left out. This was repeated 9,853 times, i.e. once for each individual in the data set, and used to obtain the mean Brier score. After the models could be prioritized with the Brier score, we performed a Bland-Altman analysis in order to assess the agreement and clinical relevance of the differences in risk score between the selected model and the original SCORE model, (see Supporting Information, Figures S3–S4).

General descriptive analyses and the fitting of survival models were performed with Stata 11 (Stata Corp, College Station, TX, USA). The actual risk calculations based on these results were obtained with R 2.14.1 (The R Foundation for Statistical Computing).

## Results

Table 1 compares the characteristics of the Swiss MONICA study population and the low-risk cohorts used to compute the ESC SCORE. The low-risk cohorts from the SCORE were recruited earlier and were followed over a shorter period of time than the MONICA sample. The age range was a bit wider in the SCORE sample. The proportion of CHD based on all CVD was lower in the MONICA sample, particularly in women. Amongst MONICA participants, the proportion of smokers was lower in men but higher in women. In MONICA men and women, mean blood pressure was in the range of the SCORE countries, while total cholesterol was above the range (6.2 vs. 6.0 mmol/l). In both sexes, cholesterol ratio was higher in the MONICA than in the SCORE sample.

Table 2 shows the estimated model parameters and coefficients from the original SCORE, the recalibrated and the new model. The small values suggest that all models are able to predict mortality quite accurately. Nevertheless there are distinct relative differences. With 14107×10^−6^, the new model with total cholesterol had the lowest mean Brier score, which means that it is the most accurate model for predicting CVD risk in our sample. In order to substantiate the results provided by the Brier score, we plotted and interpreted Bland-Altman plots. They show that the variation in risk predictions between the original and the new model not only leads to different mean Brier scores. As shown in Figures S3–S4 (Supporting Information), the variation itself is also substantial and of clinically relevant.

**Table 2:** Parameters and coefficients of the three models.

	Original SCORE		Recalibrated		New	
	CHD	non-CHD	CHD	non-CHD	CVD, Total cholesterol	CVD, Cholesterol ratio
α men	−22.1	−26.7	−29.5 (−32.5; −26.4)	−33.5 (−37.2; −29.9)	−30.7 (−33.1; −28.4)	−31.5 (−33.9; −29.1)
p men	4.71	5.64	6.43 (5.68; 7.18)	7.41 (6.52; 8.30)	6.99 (6.41; 7.57)	7.05 (6.46; 7.63)
α women	−29.8	−31.0	−46.9 (−54.6; −39.3)	−44.3 (−49.7; −38.9)	−45.9 (−50.3; −41.4)	−46.0 (−50.4; −41.5)
p women	6.36	6.62	10.38 (8.55; 12.22)	9.92 (8.62; 11.22)	10.49 (9.41; 11.57)	10.40 (9.32; 11.48)
Current smoking	0.71	0.63			0.56 (0.35; 0.78)	0.53 (0.32; 0.75)
Cholesterol (mmol/l or ratio)	0.24	0.02			0.02 (−0.06; 0.10)	0.09 (0.06; 0.12)
Systolic blood pressure (mmHg)	0.018	0.022			0.01 (0.00; 0.01)	0.01 (0.00; 0.01)
Brier score (mean)	14190×10^−6^		14172×10^−6^		14107×10^−6^	14184×10^−6^

* “Low-risk” countries: Belgium (n = 10,641), Italy (n = 53,439), Spain (n = 4,701).

Figures in brackets are 95% confidence intervals, not given for the original SCORE model.

CVD: cardiovascular disease; CHD: coronary heart disease; non-CHD: non-coronary CVD.

Cholesterol ratio: Total-to-HDL(high-density lipoprotein)-cholesterol.

**Figure 1:**
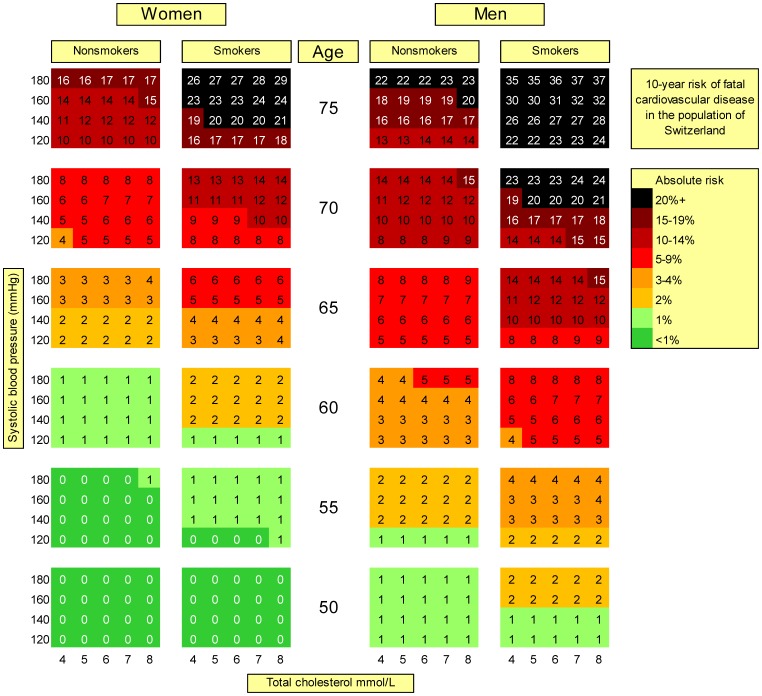
Chart for absolute 10-year risk of fatal cardiovascular disease based on the new model using total cholesterol. 9,446 participants of the Swiss MONICA study conducted 1983–92, ages 25–74 years at baseline. MONICA: MONItoring of trends and determinants in CArdiovascular disease, entire population with full follow-up Each risk percentage is calculated using a combination of given risk factor values. E.g., a man aged 65, smoker, with a systolic blood pressure of 180 and a total cholesterol of 6 mmol/L has an absolute risk (within the next 10 years) of fatal CVD of 14%.

**Figure 2:**
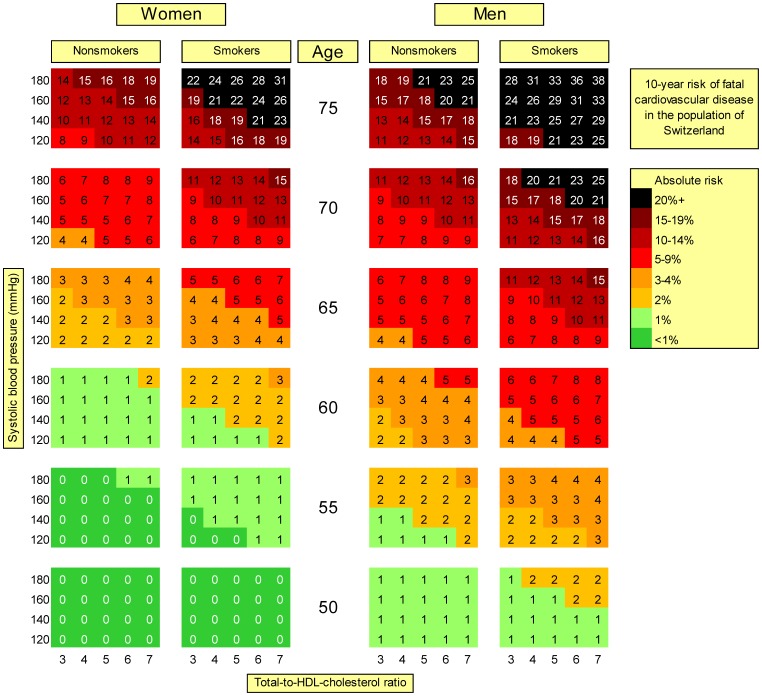
Chart for absolute 10-year risk of fatal cardiovascular disease based on the new model using cholesterol ratio. 9,446 participants of the Swiss MONICA study conducted 1983–92, ages 25–74 years at baseline MONICA: MONItoring of trends and determinants in CArdiovascular disease, entire population with full follow-up HDL: High-density lipoprotein Each risk percentage is calculated using a combination of given risk factor values. E.g., a man aged 65, smoker, with a systolic blood pressure of 180 and a total cholesterol of 6 mmol/L has an absolute risk (within the next 10 years) of fatal CVD of 14%.

Figure 1 shows the risk chart based on the new model with total cholesterol, extended to higher age categories compared to the original SCORE. In men younger than 50 years and women younger than 55 years, risks remained approximately constant. This was also the case in the recalibrated model (Supporting Information, Figure S1). Particularly in younger age classes (50–60 years), absolute risks were substantially smaller in the new model than in the recalibrated and original SCORE model (Supporting Information, Figure S2). The difference attenuated with increasing age. In contrast, the impact of total cholesterol remained consistent over all ages: there was only a minimal risk gradient over the cholesterol range (4–8 mmol/L) in the new model, while in the original and the recalibrated model the relative risk of the highest vs. the lowest cholesterol concentration ranged between 1.5 and 2.0. Still, the impact of total cholesterol was lower than that of systolic blood pressure (relative risk of 3.0 to 5.0 from 120–180 mmHg). As shown in Figure 2, the new model based on total-to-HDL cholesterol ratio (instead of total cholesterol) discriminated more strongly older persons (70+ years) at high and low risk.

## Discussion

### Main Results

Using linked data and relying on the methodology of the ESC SCORE, we evaluated how well three different models were able to predict fatal CVD events in the population of Switzerland. The new model was more appropriate than the original and the recalibrated SCORE, the latter showing however some improvement. Compared to the new model, both the original and the recalibrated models overestimated CVD risk in Switzerland. The largest variation was found in younger persons and for the impact of cholesterol, which was smaller in the new model.

### Comparison with Other Studies

To the best of our knowledge, there have been no previous attempts similar to ours. There were various efforts to adapt SCORE in order to obtain more valid estimations of CVD risk in a specific population. However, such recalibrations are limited by the fact that they rely on risk factors from persons participating in a health survey and on mortality data from the general population.[8]–[10] Information about hazard ratios (HR), i.e. the specific effect of each CVD risk factor on mortality is crucial for determination of absolute CVD risks in a given population. With recalibration, this information does not arise from the target population but still stems from a “foreign” population. When SCORE (low-risk populations) is used, recalibration studies showed slight risk underestimation for an urban population from Greece [9] and the Spanish MONICA population [8] and a small overestimation for an urban population from Switzerland. [10] Overestimation also occurred in Norway, where the implementation of guidelines based on SCORE would have doubled the number of persons in need of CVD medication for primary prevention. [6] Conversely, relatively small variations (small overestimation) were found comparing the original SCORE with our recalibrated model (Supporting Information, Figure S1).

### Possible Explanations for Overestimation

In the past four decades, death from CVD has markedly decreased in Switzerland. [7] To date, Switzerland is amongst the European countries with the lowest CHD and stroke mortality rates. [3] This trend may rather be due to improvements in screening and treatment of CVD and its risk factors, while the prevalence of CVD risk factors has remained stable or even slightly increased over time. [4], [19] Blood pressure and cholesterol lowering medication have increased and in-hospital mortality of acute myocardial infarction has decreased. [19], [20] The data used for risk estimation of SCORE was gathered between 1974 and 1992. Applying this “historical” data to a much more recent population is implicitly prone to overestimation, particularly in younger individuals.

Another reason for the difference between our new model and the original SCORE model could be the different follow-up time which was about twice as long in our sample. A long follow-up time could “wash out” the relationship between a risk factor and mortality: the likelihood that the individual’s risk factors change increases with increasing follow-up time.

The variation between the new and the original/recalibrated model was mainly due to cholesterol which had a much smaller impact in our sample. Using total-to-HDL-cholesterol ratio provided only a small improvement (Figure 2). As shown previously, the association of CVD death with cholesterol parameters was definitely weaker than with blood pressure. [21] We found a similar pattern (weak impact of cholesterol and consistent/strong impact of blood pressure) when using data from an older study conducted in Switzerland in 1977 (results not published). [22].

### Strengths of Our Approach

Our approach has the advantage that it not only considers prevalence of CVD risk factors and CVD mortality but also includes relative CVD risks (i.e. HR). The latter may strongly vary between European countries. [5] Relative risks allow for a more appropriate estimation of CVD mortality in a specific sample because they take into account population specific variations associated with risk factors and variations over age and between sexes. Moreover, up-to-date data can be obtained with comparably little effort. A CVD cohort would be much more costly and would require 20 years or so to provide enough deaths for robust analyses. Our approach also allows continuous mortality follow-up, thus taking into account changing circumstances such as improvements in the treatment of CVD. The availability of specific HR finally enables to vary e.g. prediction age range and amount and type of selected CVD risk factors because there is no dependence on a “preset” model. Therewith, population specific aging and risk factors burden can be considered. Our linked MONICA dataset may be regarded as of relatively high quality with high completeness, participation rate and modest loss to follow-up. Also, the follow-up time was longer than in most other studies used for CVD prediction.

### Limitations of our Approach

As in most other studies, participants of the Swiss MONICA had a lower mortality (in particular CVD mortality) than the general Swiss population. [14] The representativeness of our sample was also limited because it included only regions from the French- and Italian-speaking part of Switzerland. In these regions, CVD mortality is lower than in the German-speaking part of the country. [23] We also had only one assessment of exposure at study entry and could not consider change during follow-up. Moreover, we had no information on whether individuals followed the advice that has been given upon medical examination after study inclusion, e.g. cholesterol or blood pressure lowering treatment. We have, however, no reason to assume that there were differences between either of these types of medication, e.g. regarding compliance.

### Conclusion

The Brier score based comparison of risk prediction between 1) the original ESC SCORE model, 2) a recalibrated model and 3) a new model using coefficients recalculated from the target population, showed that the new model provided the most valid CVD risk prediction. CVD risk overestimation from applying the original SCORE or the recalibrated model to our sample was mainly due to a smaller contribution of cholesterol to risk prediction in the new model. Replacing cholesterol with BMI or blood glucose could make prediction more efficient. Finally, our approach of using anonymously linked routine and observational data to predict CVD mortality risk proved to be an efficient way to obtain country specific CVD risk estimates and to minimize lag time between data collection and implementation for risk assessment in clinical practice.

## Supporting Information

Figure S1 Chart for absolute 10-year of fatal cardiovascular disease based on the recalibrated ESC SCORE model* using total cholesterol. MONICA: MONItoring of trends and determinants in CArdiovscular disease *Low risk population
(TIF)

Figure S2 Chart for absolute 10-year of fatal cardiovascular disease based on the original ESC SCORE* model using total cholesterol. *Low risk population
(TIFF)

Figure S3 **Bland-Altman plot comparing the original SCORE model with the new model based on MONICA with constant limits of agreement (dotted lines).** Interpretation of the Bland-Altman plots (S3 and S4) Figure S3 shows a Bland-Altman plot allowing to compare risk estimates obtained from the original SCORE model by Conroy et al. [1] with risk estimates from the new model based on total cholesterol from MONICA. [14] For each individual, the plot shows the *mean* of the two estimated risks to be compared on the x-axis and the *difference* between the two individual risk estimates on the y-axis. This allows the detection of patterns in the differences between the two models for risk prediction. If most differences lie between the so-called limits of agreement (dotted lines) and this range of differences has no clinical relevance, it means that the two methods lead to similar predictions and are thus exchangeable. This is obviously not the case in our comparison.
(TIF)

Figure S4 **Bland-Altman plot Bland-Altman plot comparing the original SCORE model with the new model based on MONICA with limits of agreement depending on the mean difference of the risks (dotted lines).** Interpretation of the Bland-Altman plots (S3 and S4). In our case, the mean difference between both methods (solid line) is close to zero, but many differences are outside the limits of agreement. The magnitude of the differences increases with the size of the individual mean measurement, for which reason the assumption of constant limits of agreement seems to be inappropriate. Instead, the limits of agreement have to be dependent of the mean difference of the risks, which can be seen in Figure S4: Almost all points lie within the limits of agreement, however, the depicted differences in predicted risks between the two models are - especially for higher risks - much too large to be ignored. For this reason, the two methods don't seem to be comparable. The Bland-Altman plots thus underline the results obtained with the Brier score suggesting that there are substantial differences in predictions from the original and the new model.
(TIF)
