# Supplementary figures and images for: Population Specific and Up to Date Cardiovascular Risk Charts Can Be Efficiently Obtained with Record Linkage of Routine and Observational Data
Source: PLoS One. 2013 Feb 14;8(2):e56149. doi: 10.1371/journal.pone.0056149 (PMC3573036; doi:10.1371/journal.pone.0056149)

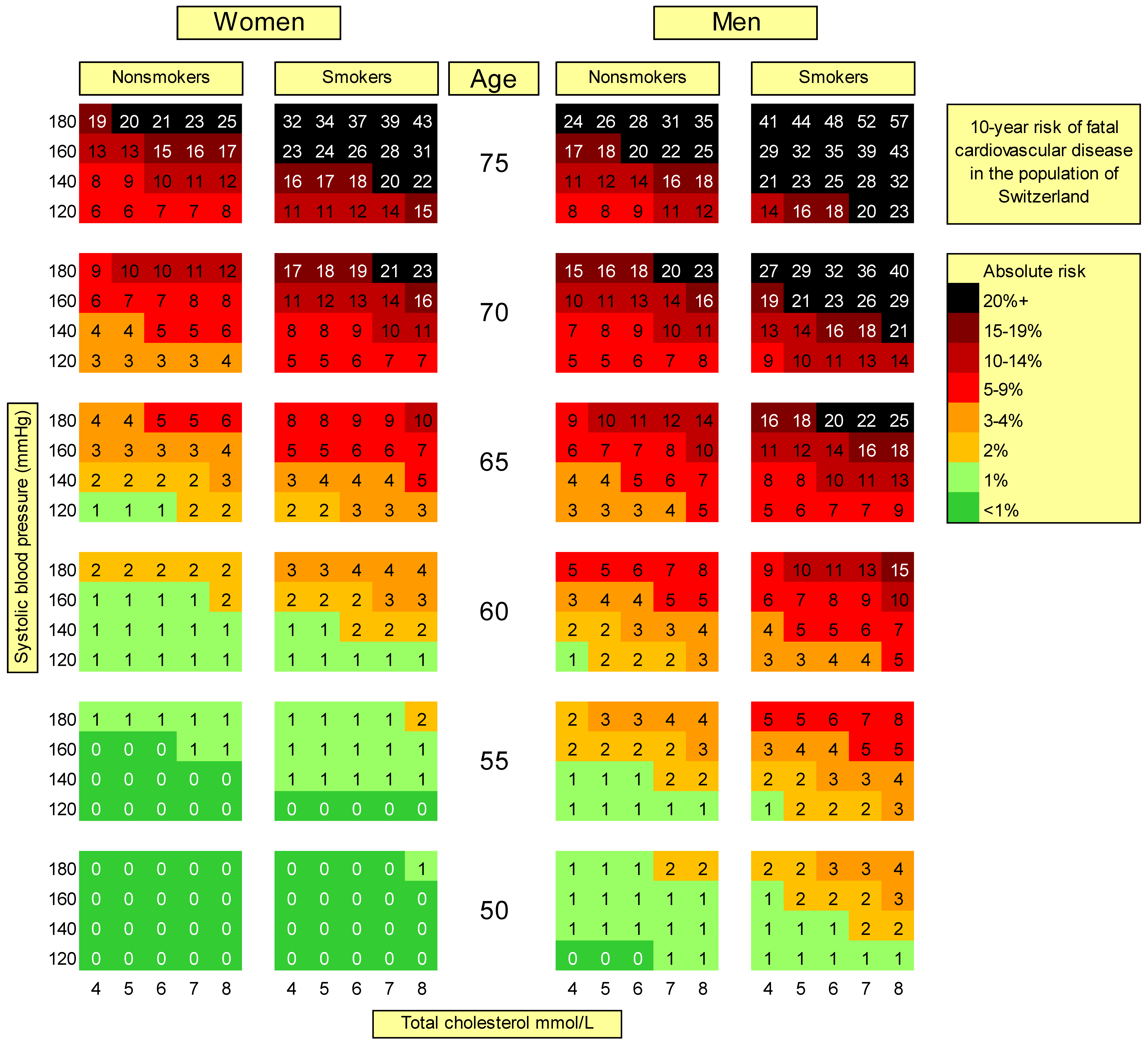

Supplement: Figure S1 — Chart for absolute 10-year of fatal cardiovascular disease based on the recalibrated ESC SCORE model* using total cholesterol. MONICA: MONItoring of trends and determinants in CArdiovscular disease *Low risk population (TIF) [file pone.0056149.s001.tif]

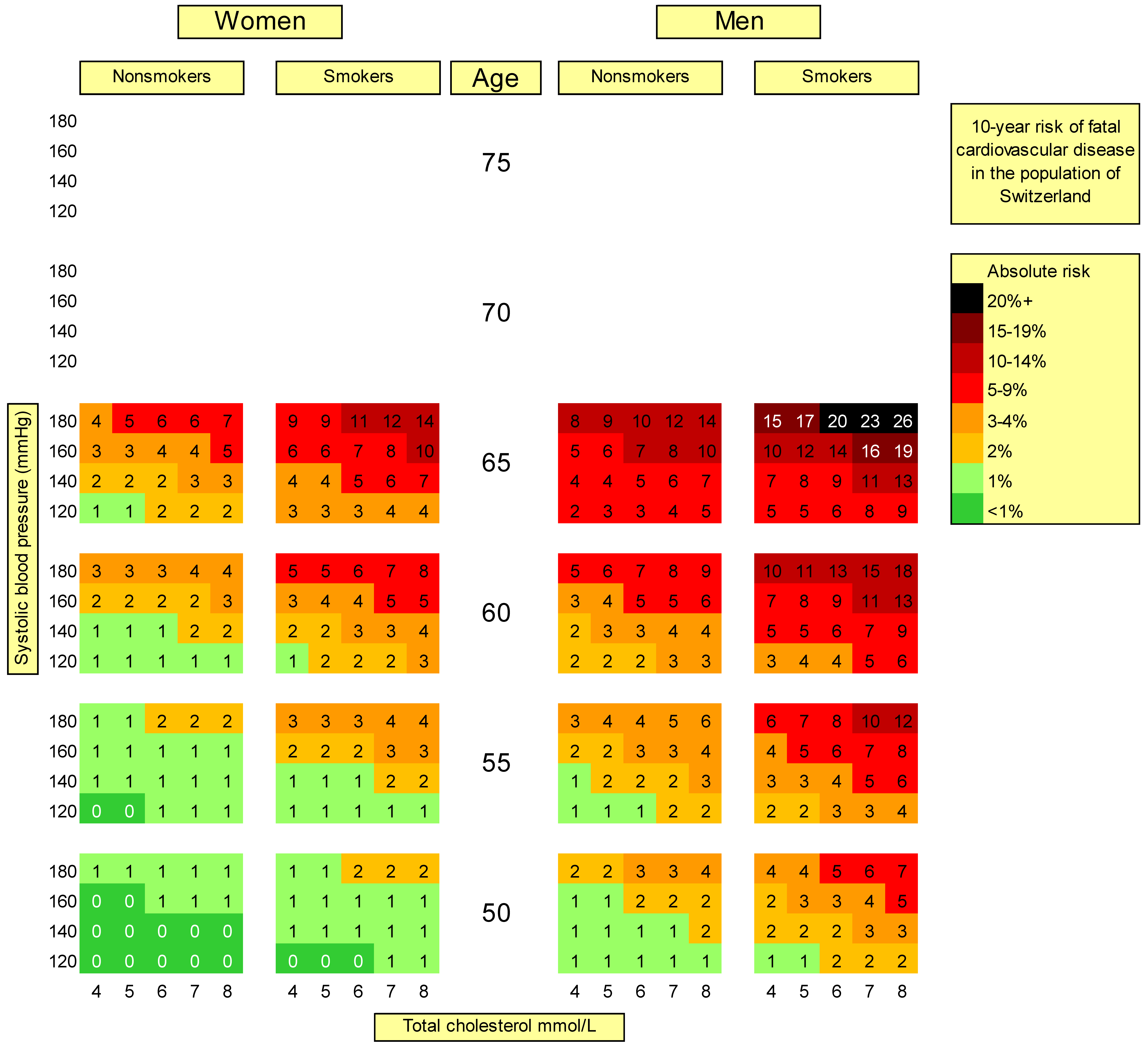

Supplement: Figure S2 — Chart for absolute 10-year of fatal cardiovascular disease based on the original ESC SCORE* model using total cholesterol. *Low risk population (TIFF) [file pone.0056149.s002.tif]

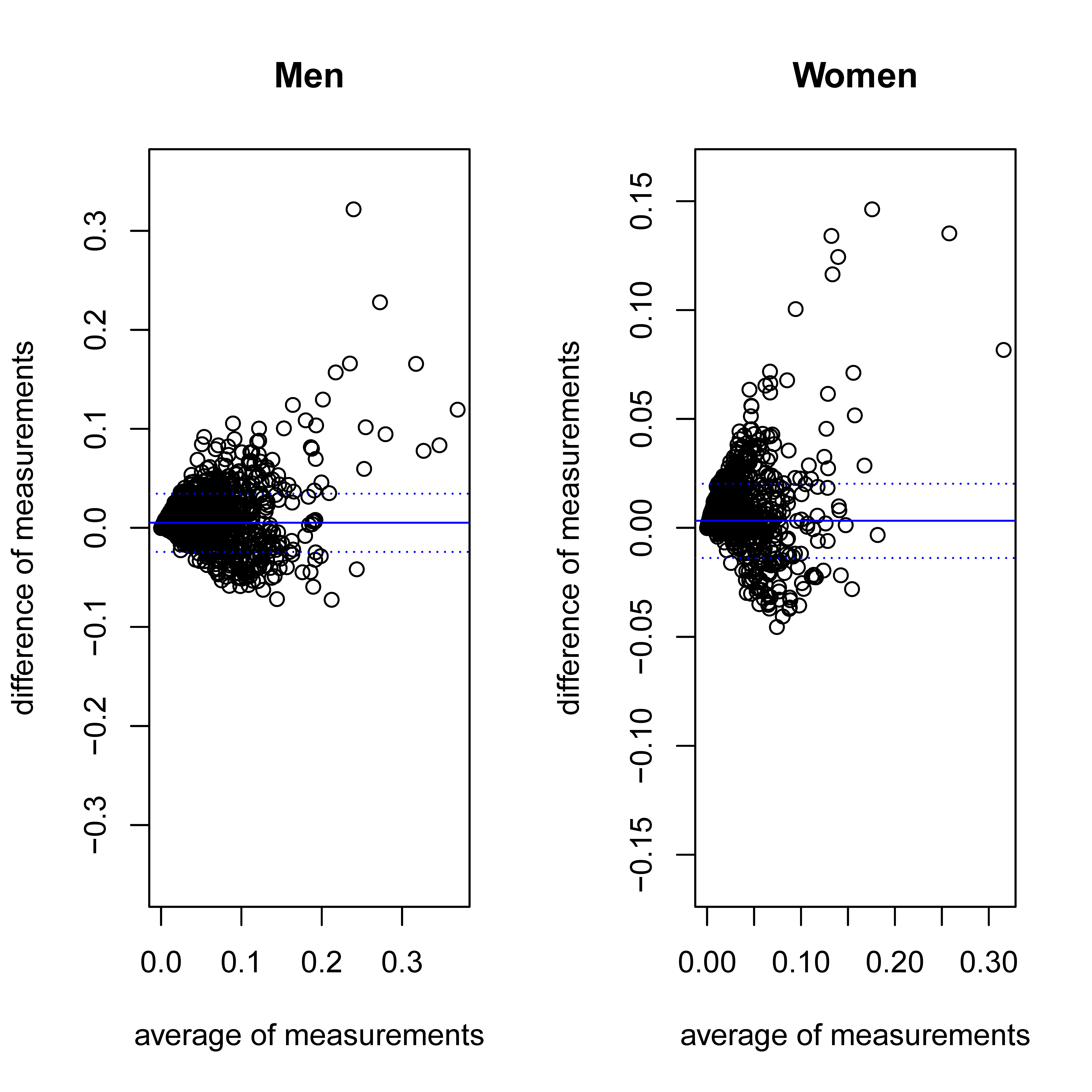

Supplement: Figure S3 — Bland-Altman plot comparing the original SCORE model with the new model based on MONICA with constant limits of agreement (dotted lines). Interpretation of the Bland-Altman plots (S3 and S4) Figure S3 shows a Bland-Altman plot allowing to compare risk estimates obtained from the original SCORE model by Conroy et al. [1] with risk estimates from the new model based on total cholesterol from MONICA. [14] For each individual, the plot shows the mean of the two estimated risks to be compared on the x-axis and the difference between the two individual risk estimates on the y-axis. This allows the detection of patterns in the differences between the two models for risk prediction. If most differences lie between the so-called limits of agreement (dotted lines) and this range of differences has no clinical relevance, it means that the two methods lead to similar predictions and are thus exchangeable. This is obviously not the case in our comparison. (TIF) [file pone.0056149.s003.tif]

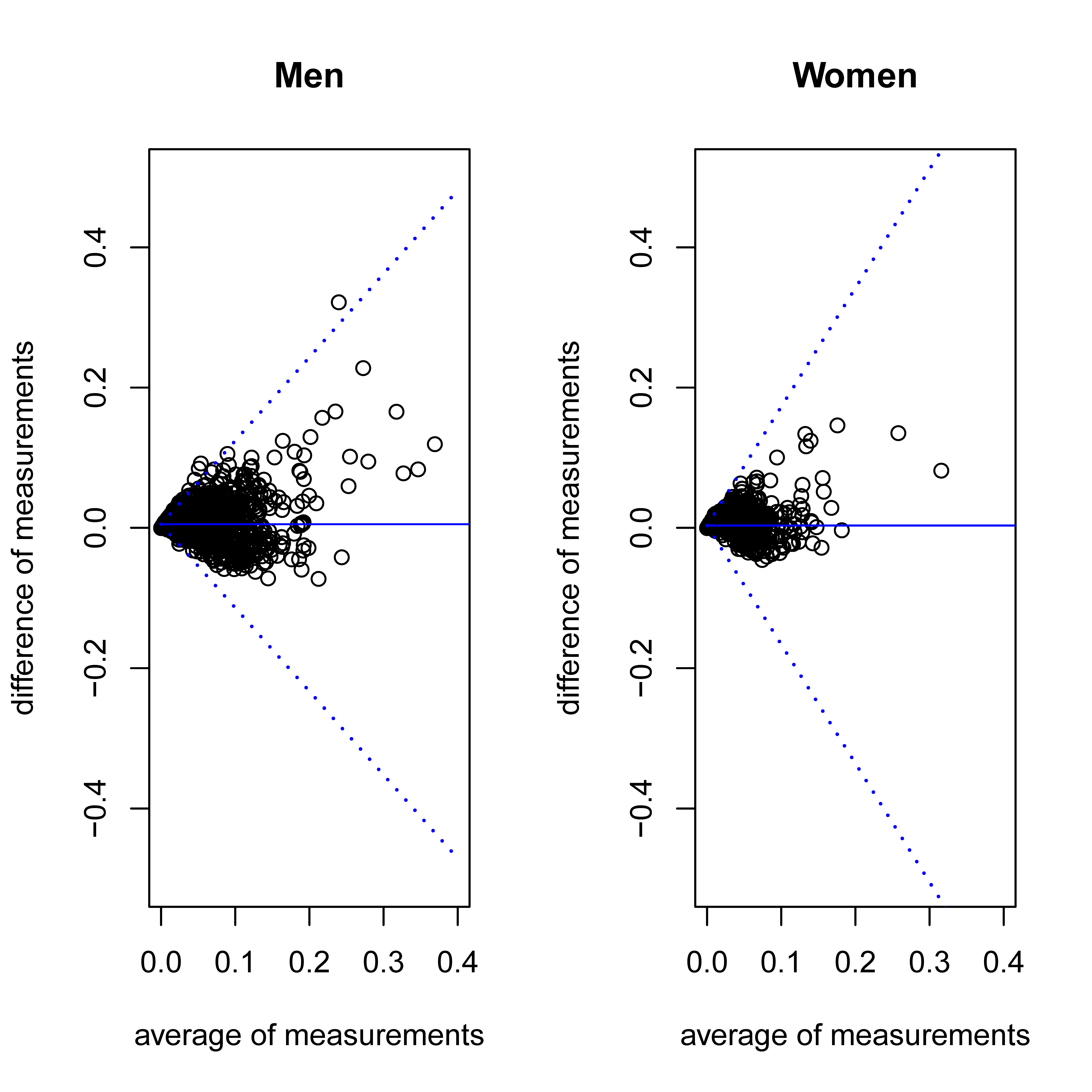

Supplement: Figure S4 — Bland-Altman plot Bland-Altman plot comparing the original SCORE model with the new model based on MONICA with limits of agreement depending on the mean difference of the risks (dotted lines). Interpretation of the Bland-Altman plots (S3 and S4). In our case, the mean difference between both methods (solid line) is close to zero, but many differences are outside the limits of agreement. The magnitude of the differences increases with the size of the individual mean measurement, for which reason the assumption of constant limits of agreement seems to be inappropriate. Instead, the limits of agreement have to be dependent of the mean difference of the risks, which can be seen in Figure S4: Almost all points lie within the limits of agreement, however, the depicted differences in predicted risks between the two models are - especially for higher risks - much too large to be ignored. For this reason, the two methods don't seem to be comparable. The Bland-Altman plots thus underline the results obtained with the Brier score suggesting that there are substantial differences in predictions from the original and the new model. (TIF) [file pone.0056149.s004.tif]
